# Scopolamine-Induced Memory Impairment in Mice: Neuroprotective Effects of *Carissa edulis* (Forssk.) Valh (Apocynaceae) Aqueous Extract

**DOI:** 10.1155/2020/6372059

**Published:** 2020-08-31

**Authors:** Fanta Sabine Adeline Yadang, Yvette Nguezeye, Christelle Wayoue Kom, Patrick Herve Diboue Betote, Amina Mamat, Lauve Rachel Yamthe Tchokouaha, Germain Sotoing Taiwé, Gabriel Agbor Agbor, Elisabeth Ngo Bum

**Affiliations:** ^1^Centre for Research on Medicinal Plants and Traditional Medicine, Institute of Medical Research and Medicinal Plants Studies, P.O. Box 13033, Yaounde, Cameroon; ^2^Department of Biological Sciences, Faculty of Science, University of Ngaoundere, P.O. Box 454, Ngaoundere, Cameroon; ^3^Department of Zoology and Animal Physiology, Faculty of Science, University of Buea, P.O. Box 63, Buea, Cameroon

## Abstract

Alzheimer's disease is first characterised by memory loss related to the central cholinergic system alteration. Available drugs provide symptomatic treatment with known side effects. The present study is aimed to evaluate the properties of *Carissa edulis* aqueous extract on a Scopolamine mouse model as an attempt to search for new compounds against Alzheimer's disease-related memory impairment. Memory impairment was induced by administration of 1 mg/kg (i.p.) of Scopolamine for 7 days, and mice were treated with *Carissa edulis* aqueous extract. Behavioural studies were performed using T-maze and novel object recognition task for assessing learning and memory and open field test for locomotion. Brain acetylcholinesterase enzyme (AChE) activity was measured to evaluate the central cholinergic system. The level of MDA, glutathione, and catalase activity were measured to evaluate the oxidative stress level. Administration of Scopolamine shows a decrease in learning and memory enhancement during behavioural studies. A significant decrease in the time spent in the preferred arm of T-maze, in the time spent in the exploration of the novel object, and in the discrimination index of the familiar object was also observed. The significant impairment of the central cholinergic system was characterised in mice by an increase of AChE activity to 2.55 ± 0.10 mol/min/g with an increase in oxidative stress. Treatment with the different doses of *Carissa edulis* (62.8, 157, 314, and 628 mg/kg orally administrated) significantly increased the memory of mice in T-maze and novel object recognition tests and also ameliorated locomotion of mice in the open field. *Carissa edulis* aqueous extract treatment also decreases the AChE activity and brain oxidative stress. It is concluded that administration of *Carissa edulis* aqueous extract enhances memory of mice by reducing AChE activity and demonstrating antioxidant properties. This could be developed into a novel therapy against memory impairment related to Alzheimer's disease.

## 1. Introduction

Neurodegenerative diseases cause the deterioration of nerve cells, affect the good functioning of the nervous system, and may affect locomotion, language, perception, cognition, and memory during its evolution depending on the regions affected [1]. Dementia is a major public health problem around the world, and its rate is increasing among older people [2]. An example of dementia is Alzheimer's disease (AD) characterised by an elevation of amyloid beta (A*β*) and phosphorylated Tau protein associated with alterations of the central cholinergic system and irreversible loss of cognitive function resulting to memory deterioration [3, 4]. Today, there is still no cure for the disease or even to stop its progression. However, currently available therapeutic strategies are mainly symptomatic slowing the evolution of the disease [5]. The main treatments for the disease are acetylcholinesterase inhibitors and N-methyl-D-aspartate receptor antagonists [3]. Unfortunately, these drugs have been connected to several adverse effects like nausea, vomiting, anorexia, and insomnia, due to nonselective action on a variety of organ tissues both centrally and peripherally [6].

Acetylcholine is a neurotransmitter widely present in the central nervous system involved in brain functions such as cortical development and activity, cerebral blood flow control, sleep-wake cycles, and cognitive and memory processes [7]. The cholinergic system is one of the main neurotransmission pathways in the brain involved in memory and cognitive mechanisms [8]. The significant reduction in cholinergic activity was the first pathophysiological component identified in AD [7]. Neurodegeneration of the cholinergic neurons is accompanied by an alteration in the synthesis of acetylcholine or its presynaptic recapture which results in the progressive impairment of memory capacity [3, 7]. This information has led to the establishment of a cholinergic hypothesis of the disease as a research platform in finding lasting treatments for cholinergic deficits hence restoring the memory performance in AD patients. Scopolamine is a muscarinic receptor antagonist that blocks cholinergic neurotransmission, leading to memory impairment in Rodents. Recent studies have reported that Scopolamine increases the accumulation of reactive oxygen species that induces oxidative stress leading to memory impairment [9]. The cholinergic hypothesis can be applied by an injection of Scopolamine which induces cognitive deficits mimicking those observed in AD, and treatment will be aimed at restoring the activity of the cholinergic system by inhibiting acetylcholinesterase enzyme.

Medicinal plants are a promising source of future drugs for AD. In general, *Carissa edulis* (*C. edulis*) was selected because of its use in traditional medicine to treat dementia. *C. edulis* is a plant widely distributed in Africa and used in the management of malaria, headaches, fever, oxidative stress, and inflammatory-related disorders such as rheumatism. *C. edulis* has been the subject of several pharmacological studies including *in vitro* antioxidant activity by scavenging the DPPH and ABTS radicals [10]; anticonvulsant activity through different mechanisms such as voltage-gated sodium, calcium, and potassium or GABAergic pathway [11]; diuretic activity by increasing blood flow in the kidneys and increase in the glomerular filtration rate resulting in increased urine output [12]; antiviral activity against the Herpes simplex virus (HSV) *in vitro* and *in vivo* studies [13] and antiplasmodial activity against the chloroquine-sensitive strains of *Plasmodium falciparum* parasite [14]; and hepatoprotective effect against subchronic administration of dimethoate on guinea pigs by normalising and restoring the liver enzyme and the antioxidant markers [15]. In all these, *C. edulis* extracts have been reported to be well tolerated in experimental animals even at high dose levels of 5000 mg/kg [16–18]. In order to search for safe and new compounds against memory impairment related to Alzheimer's disease, the present study is aimed at evaluating the neuroprotective and the memory enhancement effects of *C. edulis* on Scopolamine-induced memory impairment and oxidative stress in mice.

## 2. Material and Method

### 2.1. Plant Material

The leaves of *C. edulis* were harvested in the Far North region of Cameroon, and the identification was confirmed at the National Herbarium Yaounde-Cameroon where the voucher specimen was kept under the number 2965/SRFK. The leaves were washed, dried in the shade, crushed, and sieved to obtain a fine powder.

### 2.2. Preparation of Aqueous Extract

Ten grams of the powdered plant material was introduced into a beaker containing 60 ml of distilled water and boiled for 20 minutes. After cooling, the mixture was filtered using Wattman paper no. 1. The filtrate obtained (*C* = 62.8 mg/ml) was diluted with distilled water at 1/10, 1/4, and 1/2 and administered to the mice in a volume of 10 ml/kg. To calculate the amount of dry matter in the aqueous extract, the *C. edulis* aqueous extract earlier prepared as above (35 ml) of the filtrate was evaporated in an oven (80°C) over 24 hours, and 2.2 g of dry extract was obtained. The different doses prepared in distilled water were 62.8, 157, 314, and 628 mg/kg per 10 ml/kg of body weight.

### 2.3. Animals

Thirty-five mice *Mus musculus* Swiss of 2 months old weighing between 25 and 30 g were obtained from the animal house of the Institute of Medical Research and Medicinal Plants Studies, Yaounde, Cameroon. All these animals were kept in plexiglass cages, in an environment with an ambient temperature of about 25°C, and under a cycle of 12 hours of light and 12 hours of darkness. Two days before the experiments, the animals were progressively deprived of food to maintain them at 80-85% of their body weight. All experiments were handled in accordance with the internationally accepted principles for laboratory animal use and care.

### 2.4. Drugs and Chemicals

Scopolamine hydrobromide, Donepezil, acetylthiocholine iodide, 5,5′-dithiobis (2-nitrobenzoic acid) (DTNB), thiobarbituric acid, trichloroacetic acid, and hydrogen peroxide were obtained from Sigma-Aldrich.

### 2.5. Experimental Design

The principle is to induce memory impairment in mice by intraperitoneal injection (i.p.) of Scopolamine (1 mg/kg), which is a selective muscarinic acetylcholine receptor antagonist, and to evaluate behavioural and biochemical effects on mice. The animals were divided into 7 groups of 5 mice each: control group receiving distilled water, Scopolamine group (Scopo) receiving only Scopolamine (1 mg/kg i.p.), 4 test groups of aqueous extract receiving the *C. edulis* aqueous extract at different doses (62.8, 157, 314, and 628 mg/kg) followed by the injection of Scopolamine (1 mg/kg i.p.), and a positive control group receiving Donepezil (5 mg/kg) followed by the injection of Scopolamine (1 mg/kg i.p.). Donepezil and *C. edulis* aqueous extract were administrated orally 30 minutes before Scopolamine injection. All the treatments were daily administered for 7 consecutive days.

### 2.6. Behavioural Studies

#### 2.6.1. T-Maze Test

During the habituation phase, the animals were placed in the T-maze to familiarise with the apparatus for a period of 5 minutes. The first arm chosen by the animal was noted preferred arm and the other noted discriminated arm. During the acquisition phase, 24 hours later, the discriminated arm by the animal was closed, and the food was placed in the preferred arm. The mouse was placed in the starting arm and allowed to move to the open arm. This exercise was done for 5 minutes for each mouse. During the retention phase, the control group and the Scopolamine group were treated with distilled water, the positive control group by Donepezil (5 mg/kg), and the four test groups by the different doses of the aqueous extract (62.8, 157, 314, and 628 mg/kg). Thirty (30) minutes after the different treatments, all the groups received Scopolamine (1 mg/kg) intraperitoneally except for the control which received distilled water. Thirty minutes after administration of Scopolamine, the mice were introduced one after the other into the T-maze for a period of 5 minutes. After each passage, the apparatus was cleaned with alcohol (70% ethanol), in order to eliminate as much as possible the odorous traces left by the previous mouse. The following parameters were recorded: the latency time to choose the preferred arm, the time spent in the different arms of the T-maze (preferred and discriminated), and the number of entries in the different arms (preferred and discriminated).

#### 2.6.2. Locomotion Activity in Open Field

With the aim to verify the locomotion activity of animals evaluated in the T-maze test with induction of memory impairment, locomotion activity in an open field was done. The open field represents a new stressful environment for the animal and allows for evaluation of locomotor activity, level of exploration, and emotional response in animals [19], in a single day. Animals that received Scopolamine and were analysed for memory in the T-maze were immediately placed in the open field. The following parameters were noted for a period of 5 minutes for each mouse: the number of “crossing” (number of crossed lines or crossed tiles), the number of “rearing” (when the animal is placed on its hind legs by resting on the wall of the device with its front legs), and the time spent in the centre [19].

#### 2.6.3. Object Recognition Task in Open Field

The object recognition test was performed using the method described by Ennaceur and Delacour [20]. This test was conducted in an open field box (50 × 50 × 40 cm) and is comprised of three phases. At the end of the treatment, the mice were allowed to explore the open field for 5 min during the habituation phase. During the acquisition phase (T1), two identical objects (red cubes 4 × 4 × 4 cm) were placed in two corners of the open field at 10 cm from the sidewall. The mice were placed in the middle of the open field and allowed to explore these two identical objects for 5 minutes. After, they were put back in their cages. Subsequently, 24 hours after T1, the test “choice” (T2) was made. During T2, a new object (blue cone) has been introduced and mice were reexposed to the two objects: the familiar (F) and the new (N). The time spent by the mice in the exploration of each object during T1 and T2 was manually recorded using a stopwatch. A discrimination index (DI) was then calculated as follows:

(1) $$\begin{array}{c} \text{DI}=\frac{\left(\text{TN}-\text{TF}\right)}{\left(\text{TN}+\text{TF}\right)}\times 100. \end{array}$$

We used the discrimination index (DI) to evaluate the curiosity of mice towards new objects. The DI was defined as the percentage of time spent on a novel object to the total time spent on familiar and novel objects, where TN is time spent with the novel object and TF is time spent with the familiar object.

### 2.7. Tissue Preparation

Immediately after the behavioural tests, the mice were sacrificed by cervical decapitation, and then, the brains were quickly removed and placed in boxes containing frozen saline for its solidification for 10 min. The brain was introduced into a graduated cylinder followed by the addition of PBS (pH 7.4) to obtain the 10% homogenate. Each tube was centrifuged at 10 000 rpm/min at 4°C for 15 minutes, and the supernatant was collected for biochemical analyses.

### 2.8. Biochemical Analysis

#### 2.8.1. Estimation of Malondialdehyde

A MDA assay was performed according to the protocol described by Wilbur et al. [21], with some modifications. For this assay, 500 ml of homogenate was introduced in the test tubes and 500 *μ*l of Tris-HCl buffer (50 mM, pH 7,4 ) in the control tube. In each tube, 250 ml of trichloroacetic acid (TCA) 20% and 500 *μ*l of TBA 0.67% were added. The tubes were closed with glass beads and then incubated in water bath for 10 min at 90°C. They were then left in room temperature for cooling before being centrifuged at 3000 rpm for 15 minutes. The supernatant was piped, and the absorbance was read with a spectrophotometer at 530 nm against the control. The concentration of MDA in mol/g was determined using the formula of Beer-Lambert using the molar extinction coefficient 1.56 × 10^5^ mmol^−1^ cm^−1^ [22].

#### 2.8.2. Estimation of Catalase Activity

The activity of catalase was measured by the method of Aebi [23]. In 0.1 ml of supernatant, 1.9 ml of 50 mM phosphate buffer (pH 7.0) was added. The reaction was initiated by the addition of 1.0 ml of newly prepared 30 mM H_2_O_2_. The rate of decomposition of H_2_O_2_ was measured by spectrophotometry from absorbance changes at 240 nm. Catalase activity was expressed in units/mg protein.

#### 2.8.3. Estimation of Reduced Glutathione (GSH)

The reduced glutathione (GSH) was assessed in the brain supernatant using Ellman's reagent as described by Ellman [24]. Twenty (20) *μ*l of brain homogenates was mixed with 3 ml of Ellman's reagent at room temperature. After one hour, the absorbance of the yellow compound was read at 412 nm using a spectrophotometer. The amount of glutathione was calculated with the formula of Beer-Lambert using the extinction coefficient value of 13.600 mol^−1^ cm^−1^.

#### 2.8.4. Estimation of Acetylcholinesterase Activity

The amount of acetylcholinesterase was estimated by the method described by Ellman [25]. For the estimation of the acetylcholinesterase activity, 20 *μ*l of 0.1 M Tris-HCl buffer (pH 8.0) is introduced, 3 ml of Ellman's reagent (DTNB) in all tubes (blank and assays), and 100 *μ*l of brain homogenate in test tubes. After rapid homogenization at room temperature, the absorbance was read at 412 nm after 30 sec and 90 sec against the blank.

#### 2.8.5. Total Protein Concentration

Quantification of total proteins in mouse brain homogenates was performed according to the protocol of the Human Diagnostics Worldwide Total Protein Kit.

### 2.9. Statistical Analysis

Data were analysed using GraphPad Prism software version 5.0. The data was presented as the mean ± SEM (*n* = 5). For normally distributed data, comparison among the different studied groups was done using one-way ANOVA followed by the Tukey multiple comparison test. The Scopolamine group was analysed compared to the control group, and the groups receiving the extract were compared to the Scopolamine group. The difference was taken to be statistically significant at *p* < 0.05.

## 3. Results

### 3.1. Effects of *C. edulis* on Scopolamine-Induced Memory Impairment in the T-Maze Test

#### 3.1.1. Latency Time

**Figure 1:**
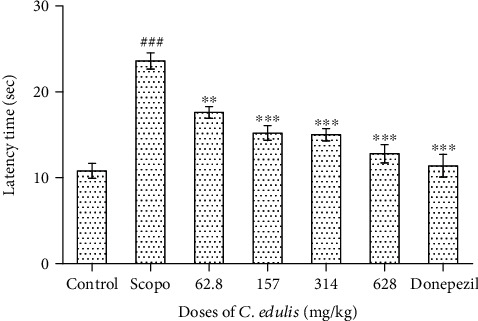
Latency time to enter into the preferred arm of the T-maze. Each bar represents the mean ± SEM (*n* = 5). The latency time increases for the Scopolamine group (^###^*p* < 0.001 vs. control), and the administration of *C. edulis* aqueous extract and Donepezil decreases this latency time (^∗∗∗^*p* < 0.001 vs. Scopo). One-way ANOVA followed by the Tukey multiple comparison test. Scopo: Scopolamine.

The effect of the aqueous extract of *Carissa edulis* on the latency time for the mice to enter the preferred arm in the T-maze is shown in Figure 1. The results show a significant increase (*p* < 0.001) of the latency time of entry into the preferred arm in the Scopolamine group compared to the control group. This latency time significantly decreases (*p* < 0.001) when the animals are treated with the different doses of *C. edulis* and Donepezil compared to the Scopolamine group from 23.6 ± 1.14 sec to 12.8 ± 1.30 sec for the mice receiving the dose of 628 mg/kg of *C. edulis* extract and 11.4 ± 1.43 sec for those receiving Donepezil used as a positive control. These times are comparable to the control group that received no treatment with a latency time of 10.8 ± 0.83 sec.

#### 3.1.2. Time Spent in the Different Arms of the T-Maze

**Figure 2:**
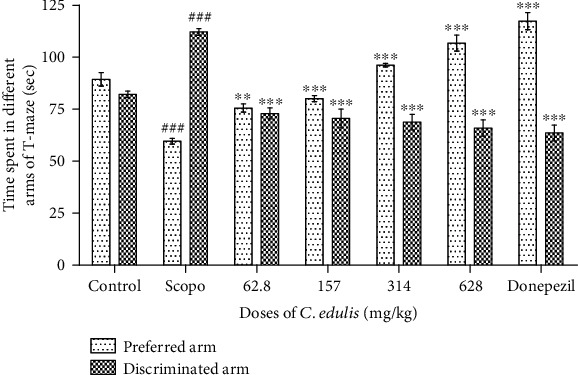
Time spent in the preferred and discriminated arms of the T-maze. Each bar represents the mean ± SEM (*n* = 5). The time spent in the preferred arm decreases, and the time spent in the discriminated arm increases in the Scopolamine group (^###^*p* < 0.001 vs. control), and the administration of *C. edulis* aqueous extract increases the time spent in the arm preferred and decreases the time spent in the discriminated arm (^∗∗^*p* < 0.01 and ^∗∗∗^*p* < 0.001 vs. Scopo). One-way ANOVA followed by the Tukey multiple comparison test. Scopo: Scopolamine.

Figure 2 shows the effects of *C. edulis* aqueous extract on the time spent in the different arms in the T-maze, the preferred and discriminated arms by the animal during the retention phase. The time spent in the preferred arm increases significantly (*p* < 0.01, *p* < 0.001) in the group of mice receiving the doses of 62.8 mg/kg, 157 mg/kg, 314 mg/kg, and 628 mg/kg *C. edulis* aqueous extract and 5 mg/kg Donepezil, respectively, in the order of 75.6 ± 4.4 sec, 80.2 ± 3.2 sec, 96.2 ± 1.7 sec, 106.8 ± 8.5 sec, and 117.4 ± 9.1 sec compared to the Scopolamine group which only passed 59.6 ± 3.20 sec. Hence, Scopolamine significantly (*p* < 0.001) reduced the time compared to the control group for the preferred arm. The opposite effect is observed in the time spent in the discriminated arm where Scopolamine significantly increased the time spent and the plant extract showing an inhibitory effect resulting to a decrease (*p* < 0.001) in all groups of mice treated with *C. edulis* aqueous extract and the Donepezil.

#### 3.1.3. Number of Entries in the Different Arms of T-Maze

**Figure 3:**
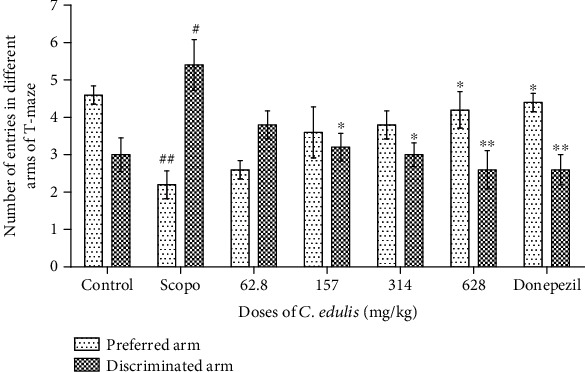
Number of entries in preferred and discriminated arms of the T-maze. Each bar represents the mean ± SEM (*n* = 5). The number of entries into the preferred arm decreases and in the discriminated arm increases in the Scopolamine group (^#^*p* < 0.05, ^##^*p* < 0.01 vs. control), and the administration of *C. edulis* increases the number of entries into the preferred arm and decreases the number of entries into the discriminated arm (^∗^*p* < 0.05 and ^∗∗^*p* < 0.01 vs. Scopo). One-way ANOVA followed by the Tukey multiple comparison test. Scopo: Scopolamine.

Figure 3 shows the number of alternations of entries into the arms of T-maze. The Scopolamine group shows a significant decrease in the number of entries into the preferred arm (*p* < 0.01) and a significant increase of entries into the discriminated arm (*p* < 0.05) compared to the control. Treatment with the dose of 628 mg/kg *C. edulis* aqueous extract as well as Donepezil resulted in an increase in the number of entries (*p* < 0.05) into the preferred arm compared to the Scopolamine group. In the discriminated arm, a significant decrease (*p* < 0.05, *p* < 0.01) in the number of entries was observed following treatment with the doses of 157 mg/kg, 314 mg/kg, and 628 mg/kg *C. edulis* aqueous extract compared to the Scopolamine group.

### 3.2. Locomotion Activity in Open Field

Immediately after the T-maze test, the mice were evaluated for their locomotor activity in the open field due to the administration of Scopolamine.  Table 1 shows a significant increase (*p* < 0.001) of the locomotor activity by the number of crossing in the mice receiving the doses of *C. edulis* aqueous extract and receiving Donepezil compared to the Scopolamine group. The crossing number of the Scopolamine group (50.8 ± 3.1) significantly decreased compared to the control (*p* < 0.001). It is the same for the rearing which sees its activity increase in the animals receiving the dose of 628 mg/kg *C. edulis* aqueous extract (*p* < 0.05) contrary to the Scopolamine group (*p* < 0.001) that decreases. The time spent in the centre of the open field decreased significantly in animals given Scopolamine (*p* < 0.001). An increase of this time was observed when the animals were treated with a dose of 628 mg/kg of *C. edulis* aqueous extract (*p* < 0.01) and Donepezil (*p* < 0.001) compared to the Scopolamine group.

### 3.3. Effects of *C. edulis* on Scopolamine-Induced Memory Impairment in Novel Object Recognition Test

#### 3.3.1. Latency Time

**Figure 4:**
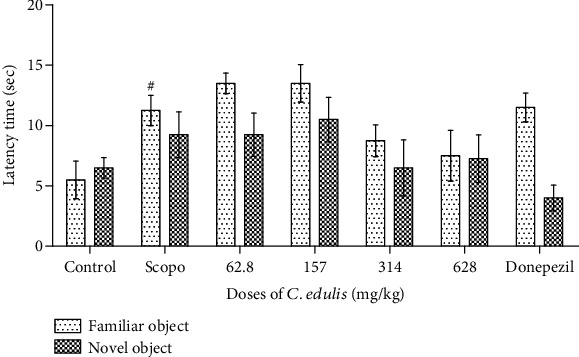
Effects of *Carissa edulis* aqueous extract on the latency time to discover the different objects. Each bar represents the mean ± SEM (*n* = 5). Latency time increases in the Scopolamine group (^#^*p* < 0.05 vs. control). No significant difference compared to the Scopolamine group. One-way ANOVA followed by the Tukey multiple comparison test. Scopo: Scopolamine.

Figure 4 shows a significant increase in the latency time to discover the familiar object F (*p* < 0.05) for the Scopolamine group compared to the control group. The administration of *C. edulis* aqueous extract decreases this latency to discover the novel object at the doses of 314 and 628 mg/kg but is not significant.

#### 3.3.2. Exploration Time of Different Objects

**Figure 5:**
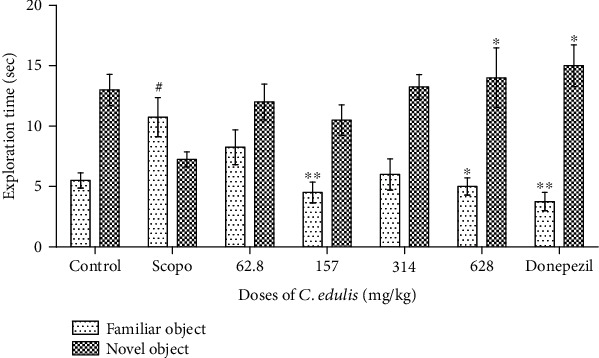
Effects of *Carissa edulis* aqueous extract on the exploration time of different objects. Each bar represents the mean ± ESM (*n* = 5). Exploration time increases in the Scopolamine group (^#^*p* < 0.001 vs. control), and administration of *C. edulis* increases this exploration time (^∗^*p* < 0.05 and ^∗∗^*p* < 0.01 vs. Scopo) for the new object. One-way ANOVA followed by the Tukey multiple comparison test. Scopo: Scopolamine.

Figure 5 shows a significant increase (*p* < 0.05) of the time spent with the familiar object F for the Scopolamine group compared to the control group. There was a significant increase (*p* < 0.05) in the time spent with the new object N at doses of 628 mg/kg *C. edulis* aqueous extract and a reduction of this time (*p* < 0.05) with the familiar object in the mice treated with the doses of 157 and 628 mg/kg of *C. edulis* aqueous extract compared to the Scopolamine group. The group receiving Donepezil showed a significant increase in time spent with the new object N (*p* < 0.05).

#### 3.3.3. Number of Explorations of the Different Objects

**Figure 6:**
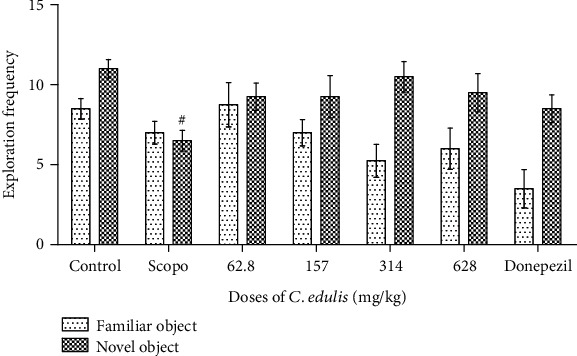
Effects of *Carissa edulis* aqueous extract on the number of explorations of different objects. Each bar represents the mean ± SEM (*n* = 5). The number of explorations decreases for the Scopolamine group (^#^*p* < 0.05 vs. control). No significant difference when compared to the Scopolamine group. One-way ANOVA followed by the Tukey multiple comparison test. Scopo: Scopolamine.

The number of visits to different objects is presented in Figure 6. There is a significant decrease of the number of explorations of the novel object for the Scopolamine group compared to the control group and an increase of this number for the new object at the different doses of *C. edulis* aqueous extract and also for Donepezil compared to the Scopolamine group, although this increase is not significant.

#### 3.3.4. Discrimination Index

**Figure 7:**
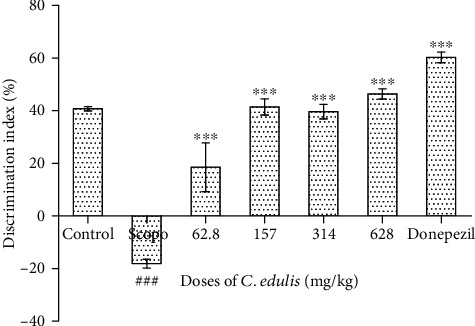
Effect of *Carissa edulis* aqueous extract on the discrimination index. Each bar represents the mean ± ESM (*n* = 5). The discrimination index decreases in the Scopolamine group (^###^*p* < 0.001 vs. control), and the administration of *C. edulis* aqueous extract increases this discrimination index (^∗∗∗^*p* < 0.001 vs. Scopo). One-way ANOVA followed by the Tukey multiple comparison test. Scopo: Scopolamine.

Figure 7 shows the results of the discrimination index of the objects. We observed a decrease of the discrimination index of the familiar object which is −18.97 ± 3.33% for the mice in the Scopolamine group compared to the control group with 40.79 ± 1.61%. A significant increase (*p* < 0.001) of this discrimination index was observed in the groups of animals receiving *C. edulis* aqueous extract at doses of 157, 314, and 628.5 mg/kg, respectively. Donepezil, the reference molecule, also increased this index of 60.22 ± 4.01% (*p* < 0.001).

### 3.4. Effects of *C. edulis* on Parameters of Oxidative Stress, Antioxidant Enzymes, Total Proteins, and Acetylcholinesterase Activity

#### 3.4.1. Level of Malondialdehyde and Antioxidant Enzymes

The administration of Scopolamine induces oxidative damage in animals. The MDA level increases significantly (*p* < 0.001) in the mice of the group receiving only Scopolamine of 105.46 ± 2.67 mmol/g of protein compared to the control group which has a level of 63.61 ± 3.56 mmol/g of protein. This level of MDA significantly decreases in the groups of animals that received the treatments with the doses of *C. edulis* aqueous extract which significantly reduces this rate to normal values compared to the control group. Scopolamine significantly (*p* < 0.05) decreased glutathione levels and catalase activity in the group treated with Scopolamine alone compared to the control group. The mice treated with *C. edulis* aqueous extract show a significant increase in these antioxidant enzymes (*p* < 0.05). The activity of catalase increases significantly compared to the Scopolamine group of 108.58 ± 4.65 mmol H_2_O_2_/mg protein to 251.44 ± 32.55, 274.36 ± 8.78, and 325 ± 18.89 mmol H_2_O_2_/mg protein for the groups treated with *C. edulis* aqueous extract at the doses of 157, 314, and 628 mg/kg, respectively (*p* < 0.001). The concentration of glutathione increased significantly for the doses of 157, 314, and 628 mg/kg compared to the group receiving only Scopolamine which is 1.63 ± 0.10 mmol/g protein and reaching normal values compared to the control group. Catalase and glutathione increased significantly with the administration of Donepezil (*p* < 0.001). The results are shown in Table 2.

#### 3.4.2. Acetylcholinesterase Activity

In the present study, the results presented in Table 2 show that Scopolamine increases the activity of acetylcholinesterase (*p* < 0.05) in the brains of mice compared to the control group. Treatment with different doses of *C. edulis* aqueous extract appears to have a memory booster effect by inhibiting the elevation of acetylcholinesterase activity compared to the group receiving only Scopolamine. There is a significant (*p* < 0.05) decrease in acetylcholinesterase activity for the group treated with *C. edulis* aqueous extract.

## 4. Discussion

The present study was undertaken to investigate whether *Carissa edulis* could improve memory impairment via the cholinergic pathways. Medicinal plants are playing a significant role in the management of memory deficit and Alzheimer's disease. In this study, we have evaluated the effect of *C. edulis* on the memory function of amnesic mice in the T-maze and novel object recognition tests. Scopolamine promoted amnesia in the animals through impaired memory by blocking the muscarinic cholinergic receptors in the brain as earlier reported [26]. In the present study, chronic administration of Scopolamine to mice increased the latency time to enter in the preferred arm resulting in a reduction in time passed in the preferred arm. Administration of *C. edulis* aqueous extract decreases this latency time resulting in an increase in time passed in the preferred arm of the T-maze. The decrease in latency time indicated an improvement of memory as earlier reported [27]. The significant increase in the number of entries and the time spent in the preferred arm reflects a good functioning of the memory [28, 29]. These results show the antagonistic effects of *C. edulis* aqueous extract on the action of Scopolamine, which could be owing to the presence of bioactive substances such as polyphenols, terpenes, tannins, flavonoids, cardiac glycosides, lignans, sesquiterpenes, and coumarins that may inhibit the effects of Scopolamine and thereby improve memory loss. Many studies report that polyphenols have antioxidant capacity neutralizing free radicals by crossing the blood-brain barrier to protect the brain and nervous system. The main functions of polyphenols include improvements in memory [9]. In addition, the increase in the number of entries and the time spent in the arms, preferred and discriminated, suggests the increase of the exploration behaviour and thus the memory faculties [29].

The open field test is used to evaluate exploration behaviour and locomotor activity of mice in response to a novel environment [30]. The mice treated with Scopolamine (1 mg/kg) show a reduced number of crossing and rearing resulting in Scopolamine-altered locomotion activity. In this test, the increase in the number of “crossing,” the number of “rearing,” and the time spent in the centre for the group of mice treated with the *C. edulis* aqueous extract indicates the increase of exploration and locomotor activity. It is possible to suggest that *C. edulis* aqueous extract has memory properties, properties that could be mediated by cholinergic neurotransmission at the level of the cerebral cortex and the hippocampus [30].

Novelty in an open field has been extensively exploited in studies of behaviour and brain functions in rats and mice in neuroscience and the study of memory [31, 32]. The results of the object recognition test showed that Scopolamine increases the latency to discover a familiar object compared to a new object. It also decreased the object exploration time correlated with exploration frequency. The discrimination index decreased significantly in the group of animals receiving Scopolamine suggesting an impairment of learning and recognition process. Hence, Scopolamine as an anticholinergic agent blocks muscarinic receptors which have been reported to disrupt memory performance and learning in humans and animals [33]. Scopolamine-induced memory disorders are associated with increased oxidative stress in the brain [34, 35] characterised by an increased MDA concentration which is a harmful effect of reactive oxygen species [36]. Since the brain is composed of lipids, the effect of ROS leading to lipid peroxidation may directly lead to brain death. The results of the present study showed a collapse in the brain antioxidant defence system characterised by a higher MDA level and lower catalase and glutathione activity in the Scopolamine-treated group compared to the control group as earlier suggested by Lee et al. [37]. Treatment with *C. edulis* aqueous extract significantly improves catalase activity and glutathione levels, while the level of MDA decreased significantly. It has been previously reported that *C. edulis* possesses antioxidant potentials [10, 38, 39], especially its hydroethanolic extract by increasing the catalase activity and glutathione level and reducing the MDA level in the mouse brain [40]. It has also been reported that the aqueous extract of *C. edulis* improves the antioxidant enzymes and reduces oxidative stress in D-galactose-induced neurotoxicity and memory impairment in rats [41]. Thus, the underlying mechanism of *C. edulis* may involve the inhibition of reactive oxygen species formation by scavenging superoxide anion, hydroxyl radicals, and hydrogen peroxide.

The central cholinergic system plays a vital role in the processes of memory [8]. A dysfunction of neurons containing acetylcholine in the elderly presents cognitive deficiencies [4, 6]. Current data are consistent with Chen et al. [42], Budzynska et al. [43], and Park et al. [44], who reported that Scopolamine produces severe cholinergic deficits and increased activity of acetylcholinesterase in the hippocampus, thereby reinforcing neurodegeneration in the brain. Treatment with *C. edulis* aqueous extract significantly reduced the activity of acetylcholinesterase compared to the Scopolamine group. This result is consistent with an earlier study where the hydroethanolic extract decreased the activity of AChE [40] and enhanced the memory in different cognitive impairment models. Thus, our data suggest that the memory enhancement effects of *C. edulis* can be explained by the inhibition of acetylcholinesterase activity and the increased release of acetylcholine into the synaptic gap and its fixation on the postsynaptic receptors.

## 5. Conclusion

This present study was conducted to evaluate the protective effects of *Carissa edulis* aqueous extract on Scopolamine-induced memory loss, which is a muscarinic acetylcholine receptor antagonist. Scopolamine-induced memory and learning deficits evaluated during behavioural studies in T-maze and object recognition tests induced an increase in oxidative stress and also an increase in acetylcholinesterase activity. *C. edulis* aqueous extract administration significantly improved memory, as demonstrated in T-maze and novel object recognition tests, and the antioxidant defence system thus protecting neurons from oxidative stress against Scopolamine-induced memory loss. This study was limited to the protective effects of *C. edulis* on the cholinergic pathway by improving the cognitive impairment induced by Scopolamine. Thus, the effects of *C. edulis* on other possible mechanisms involved in the pathophysiology of AD such as glutamatergic and inflammatory pathways remain to be verified.

## Figures and Tables

**Table 1:** Number of crossing and rearing and time spent in the centre of the open field.

Parameters	Control	Scopo	Doses of *C. edulis* (mg/kg)				Donepezil
			62.8	157	314	628	
Number of crossing	80.4 ± 2.8	50.8 ± 3.1^###^	65.4 ± 2.0^∗∗∗^	69.8 ± 3.4^∗∗∗^	72.6 ± 4.7^∗∗∗^	75.6 ± 2.7^∗∗∗^	78.6 ± 1.8^∗∗∗^
Number of rearing	10.6 ± 1.9	5.8 ± 0.8^###^	6.2 ± 0.8	7.2 ± 0.8	8 ± 1.5	8.6 ± 01.1^∗^	9.4 ± 1.1^∗∗^
Time spent	5.4 ± 1.1	2.4 ± 0.8^###^	2.8 ± 0.8	3.2 ± 1.0	4.2 ± 1.1^∗^	4.8 ± 0.8^∗∗^	5.2 ± 2.0^∗∗∗^

Each value represents the mean ± SEM (*n* = 5). The number of crossing and rearing decreases for the Scopolamine group (^###^*p* < 0.001 vs. control), and the administration of *C. edulis* aqueous extract increases this number of crossing and rearing (^∗^*p* < 0.05, ^∗∗^*p* < 0.01, and ^∗∗∗^*p* < 0.001 vs. Scopo). One-way ANOVA followed by the Tukey multiple comparison test. Scopo: Scopolamine.

**Table 2:** Effects of *C. edulis* on brain MDA, glutathione, catalase, AChE activity, and protein concentration of Scopolamine-treated mice.

	Doses (mg/kg)	MDA (mmol/g)	Glutathione (mmol/g)	Catalase (mmol H_2_O_2_/mg)	AChE (mol/min/g)	Protein (g/dl)
Control		63.61 ± 3.56	4.16 ± 0.36	290.56 ± 14.34	1.53 ± 0.04	5.96 ± 1.23
Scopo	1	105.46 ± 2.67^###^	1.63 ± 0.10^##^	108.58 ± 4.65^###^	2.45 ± 0.10^##^	5.85 ± 0.45
*C. edulis*	62.8	94.53 ± 5.06	2.16 ± 0.20	145.45 ± 7.80^∗∗^	2.12 ± 0.12	6.31 ± 0.67
	157	76.76 ± 3.14^∗^	3.86 ± 0.16^∗^	251.44 ± 32.55^∗∗∗^	1.78 ± 0.07^∗^	6.04 ± 0.11
	314	68.69 ± 3.87^∗∗^	4.19 ± 0.27^∗^	274.36 ± 8.78^∗∗∗^	1.47 ± 0.09^∗∗^	6.12 ± 0.23
	628	64.88 ± 4.47^∗∗∗^	4.29 ± 0.09^∗∗^	325.00 ± 18.89^∗∗∗^	1.40 ± 0.11^∗∗^	5.96 ± 0.78
Donepezil	5	60.38 ± 6.89^∗∗∗^	4.47 ± 0.12^∗∗^	423.60 ± 10.14^∗∗∗^	1.31 ± 0.08^∗∗∗^	6.29 ± 1.04

Each value represents the mean ± SEM (*n* = 5). The level of MDA increases and the antioxidant enzyme level decreases for the Scopolamine group (^##^*p* < 0.01, ^###^*p* < 0.001 vs. control), and the administration of *C. edulis* aqueous extract decreases the level of MDA by increasing the level of antioxidant enzymes (^∗^*p* < 0.05, ^∗∗^*p* < 0.01, and ^∗∗∗^*p* < 0.001 vs. Scopo). One-way ANOVA followed by the Tukey multiple comparison test. Scopo: Scopolamine.

## Data Availability

All the results presented in this study were carried out by authors, and data used as references were properly cited.
